# Supplementary figures and images for: Impact of Skin Decontamination Wipe Solutions on the Percutaneous Absorption of Polycyclic Aromatic Hydrocarbons
Source: Toxics. 2024 Sep 30;12(10):716. doi: 10.3390/toxics12100716 (PMC11511401; doi:10.3390/toxics12100716)

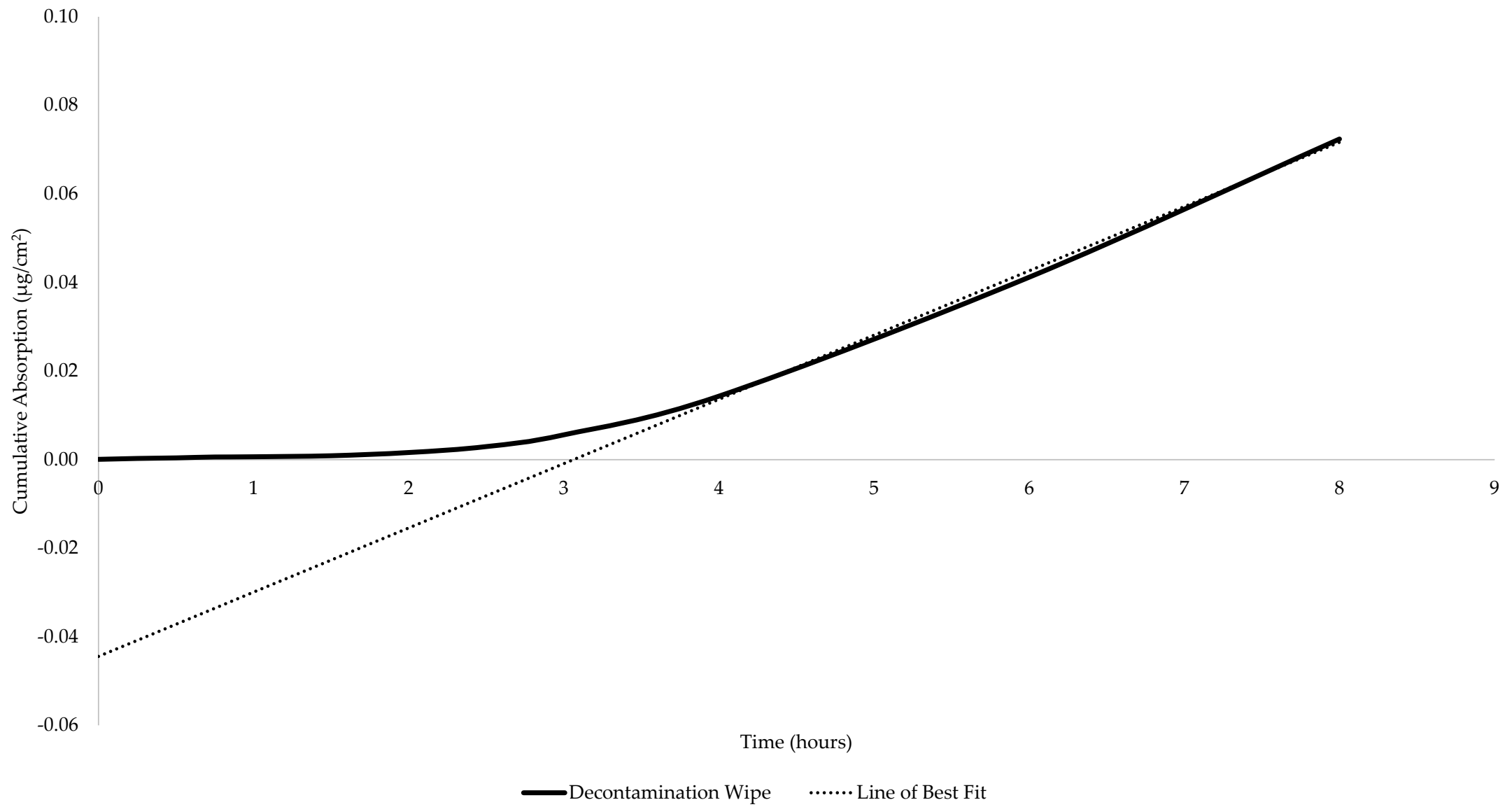

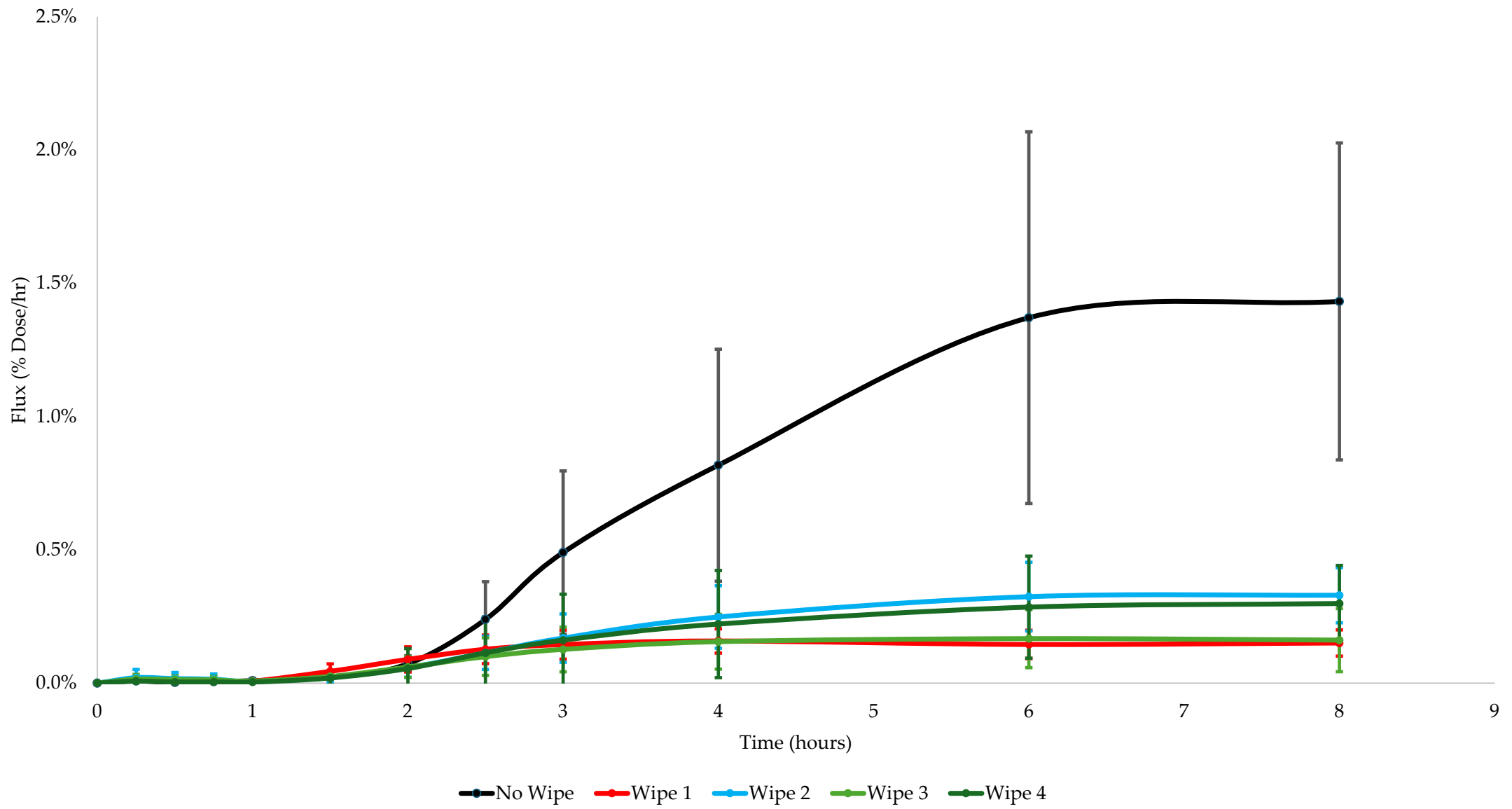

Supplement: Supplementary file 1 [file toxics-12-00716-s001.zip › Decon Wipe Effects on Dermal Absorption Data_All Figures.pdf]
